# *Lactobacillus rhamnosus *GR-1 enhances NF-kappaB activation in *Escherichia coli*-stimulated urinary bladder cells through TLR4

**DOI:** 10.1186/1471-2180-12-15

**Published:** 2012-01-22

**Authors:** Mattias Karlsson, Nikolai Scherbak, Gregor Reid, Jana Jass

**Affiliations:** 1 School of Science and Technology, Life Science, Örebro University, 701 82 Örebro, Sweden; 2 Department of Microbiology and Immunology, University of Western Ontario, London, ON N6A 5C1, Canada; 3 The Lawson Health Research Institute, St Josephs Hospital, London, ON N6A 4V2, Canada

## Abstract

**Background:**

Epithelial cells of the urinary tract recognize pathogenic bacteria through pattern recognition receptors on their surface, such as toll-like receptors (TLRs), and mount an immune response through the activation of the NF-kappaB pathway. Some uropathogenic bacteria can subvert these cellular responses, creating problems with how the host eliminates pathogens. *Lactobacillus *is a genus of lactic acid bacteria that are part of the microbiota and consist of many probiotic strains, some specifically for urogenital infections. Immunomodulation has emerged as an important mode of action of probiotic and commensal lactobacilli and given the importance of epithelial cells, we evaluated the effect of the urogenital probiotic *Lactobacillus rhamnosus *GR-1 on epithelial immune activation.

**Results:**

Immune activation through the NF-kappaB pathway was initiated by stimulation of T24 urothelial cells with heat-killed *Escherichia coli *and this was further potentiated when cells were co-cultured with live *L. rhamnosus *GR-1. Heat-killed lactobacilli were poor activators of NF-kappaB. Concomitant stimulation of bladder cells with *E. coli *and *L. rhamnosus *GR-1 increased the levels of the pro-inflammatory cytokine TNF, whereas IL-6 and CXCL8 levels were reduced. Another probiotic, *L. rhamnosus *GG, was also able to potentiate NF-kappaB in these cells although at a significantly reduced level compared to the GR-1 strain. The transcript numbers and protein levels of the lipopolysaccharide receptor TLR4 were significantly increased after co-stimulation with *E. coli *and lactobacilli compared to controls. Furthermore, inhibition of TLR4 activation by polymixin B completely blocked the lactobacilli potentiation of NF-kappaB.

**Conclusions:**

The immunological outcome of *E. coli *challenge of bladder cells was influenced by probiotic *L. rhamnosus *GR-1, by enhancing the activation of NF-kappaB and TNF release. Thus the urogenital probiotic *L. rhamnosus *GR-1 modulated the activation of the NF-kappaB through increased levels of TLR4 on the bladder cells and altered subsequent release of cytokines from urothelial cells. By influencing immunological factors such as TLR4, important in the process of fighting pathogens, lactobacilli could facilitate pathogen recognition and infection clearance.

## Background

Many bacterial diseases, including urinary tract infections (UTIs) are initiated by microorganisms adhering to and colonizing the epithelium. Epithelial cells of the urinary tract (urothelial cells) respond to pathogens by producing various immune activating substances including compounds that recruit immune cells such as macrophages. Epithelial cells express a number of different pattern recognition receptors such as toll-like receptors (TLRs) that are able to trigger the expression of inflammatory mediators and subsequent inflammation in the presence of pathogenic microbes. One of the most studied TLRs is TLR4, which binds lipopolysaccharides (LPS) found on the cell wall of Gram-negative bacteria [1]. Key proteins involved in inflammation are the Rel/Nuclear Factor (NF)-κB proteins, which once activated can induce the transcription of several immunologically essential molecules, such as tumor necrosis factor (TNF), interleukin (IL)-6 and CXCL8 [2-4]. These cytokines are very important in the antimicrobial and inflammatory process and they effectively recruit immune cells to the infected site. In its inactive form, the NF-κB transcription factor is located within the cytosol, where inhibitory proteins masking the nuclear localization signal impair its nuclear migration. During NF-κB activation, the inhibitory proteins are disassociated from the transcription factor dimer, which is subsequently transported into the nucleus [5]. Nuclear translocation of NF-κB during infectious processes is important for the subsequent activation of immune responses.

The most prevalent cause of UTI is uropathogenic *Escherichia coli *(UPEC), which expresses numerous virulence factors including toxins and fimbriae used for adhesion. Eukaryotic cells can identify pathogens, for example when type 1 fimbriae, P-pili, or LPS bind to TLR4 and elicit an inflammatory response, albeit via different intracellular pathways [6]. However, some UPEC are equipped with virulence factors that can block immune responses allowing the organisms to freely multiply. These UPEC can act directly on TLR activation, inhibiting NF-κB at an early stage after ligand binding and consequently, there is no transcription of the integral components needed to mount an immune response [7]. Similarly, a low TLR4 expression or activity is associated with increased UTI susceptibility. Such strategies would impede pathogen clearance *in vivo *and cause recurrent UTIs [8,9].

*Lactobacillus *is a genus of Gram-positive bacteria naturally found in the healthy human vagina [10] and urethra [11]. Moreover, a low *Lactobacillus *count is inversely related to high numbers of *E. coli *in the vagina and a history of recurrent UTI [12]. Several lactobacilli strains are used as probiotics to prevent infections within the gastrointestinal and urogenital tracts as well as to ameliorate allergic and inflammatory conditions [13-15]. The probiotic mechanisms are believed to include the release of antibacterial substances, biosurfactant production, disruption of biofilms and competitive exclusion [16]. Furthermore, the ability of probiotic strains to modulate immunity through NF-κB and mitogen activated protein (MAP) kinase pathways, both important in the development of innate and adaptive immunity, has been reported [17,18]. *Lactobacillus rhamnosus *GR-1 is a probiotic isolated from a female urethra [19] used to prevent UTI and bacterial vaginosis, and it has both immunomodulatory and antimicrobial activity [20,21]. Currently, the immunological effects of lactobacilli on urothelial cells are in large part unexplored. The aim of this current study was to investigate how *L. rhamnosus *GR-1 can affect urothelial immune responses to *E. coli*.

## Results

### Bladder cells responded poorly to lactobacilli compared to heat-killed *E. coli*

**Figure 1:**
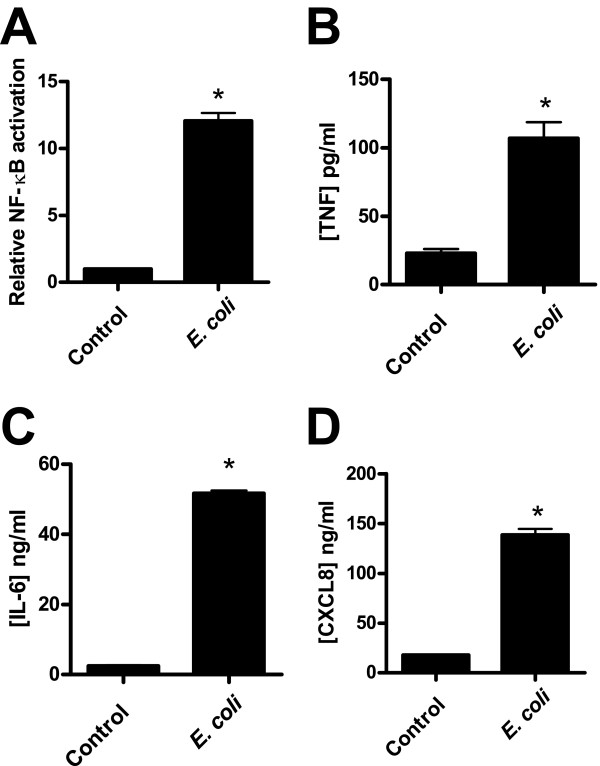
**NF-κB activation and expression of cytokines in bladder cells after *E. coli *challenge**. Bladder cells were stimulated with heat-killed *E. coli *for 24 h at a concentration corresponding to 10^8 ^cfu/ml. (**A**) Relative NF-κB activation was measured by luciferase activity (*n *= 4) and the protein levels of (**B**) TNF, (**C**) IL-6, and (**D**) CXCL8 were measured by ELISA (*n *= 3). Error bars represent the standard errors of the means. Bars labeled with an asterisk significantly differ from the control (*p*-values < 0.05).

**Figure 2:**
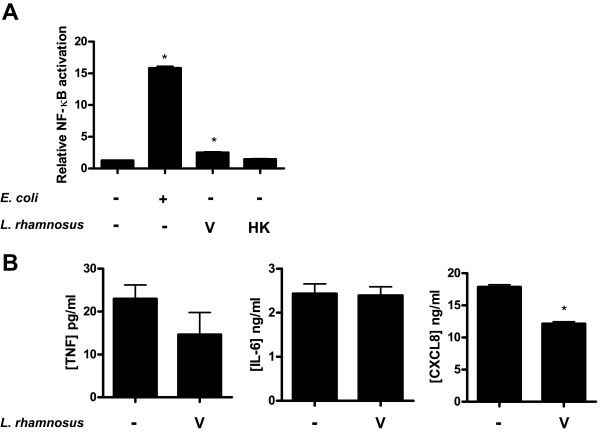
**NF-κB activation and expression of cytokines in bladder cells after stimulation with *L. rhamnosus *GR-1**. Viable (V) or heat-killed (HK) *L. rhamnosus *GR-1 at a concentration of 2 × 10^7 ^cfu/ml were used to challenge bladder cells for 24 h. (**A**) Relative NF-κB activation (*n *= 4) and (**B**) TNF, IL-6, and CXCL8 levels (*n *= 3) were measured using luciferase assay and ELISA, respectively. Error bars represent the standard errors of the means. Bars labeled with an asterisk significantly differ from the control (*p*-values < 0.05).

*E. coli *are potent activators of epithelial immune responses and were therefore used to stimulate activation of NF-κB and cytokine release from bladder cells. After 24 h of challenge with heat-killed *E. coli*, cells responded with more than 10-fold increase in NF-κB activation compared to resting cells, as measured by the luciferase reporter assay (Figure 1A). Furthermore, challenge gave a substantial increase in pro-inflammatory TNF, IL-6, and CXCL8 levels (Figure 1B, C, and 1D). On the other hand, *L. rhamnosus *GR-1 was a poor activator of NF-κB. Stimulation with viable lactobacilli led to a minor increase in the activation of NF-κB while heat-killed bacteria had no significant effect (Figure 2A). Although viable lactobacilli could marginally increase NF-κB activation compared to resting cells, stimulation did not promote release of any of the tested cytokines (TNF, IL-6 and CXCL8). In contrast, it resulted in a small but significant reduction of CXCL8, compared to resting cells, while TNF and IL-6 levels were unaffected (Figure 2B).

Lactobacilli do not normally come into contact with bladder cells, therefore we determined the cytotoxicity caused by lactobacilli exposure. However, we did not observe decreased epithelial cell viability compared to resting cells, as determined using propidium iodide stained cells and flow cytometry (data not shown).

### Viable lactobacilli potentiated NF-κB activation and cytokine response in *E. coli*-stimulated cells

**Figure 3:**
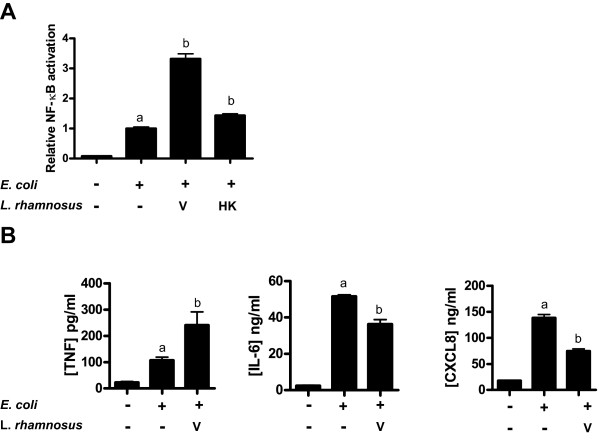
**NF-κB activation and cytokine secretion after concomitant stimulation with *E. coli *and *L. rhamnosus *GR-1**. Bladder cells were challenged for 24 h with heat-killed *E. coli *alone or together with viable (V) or heat-killed (HK) *L. rhamnosus *GR-1. (**A**) Relative NF-κB activation (*n *= 4). (**B**) TNF, IL-6 and CXCL8 levels (*n *= 3) were measured. Bars labeled "a" are significantly different from control and "b" significantly different from cells stimulated with *E. coli *(*p*-values < 0.05).

Bladder cells were relatively indifferent towards stimulation with both viable and heat-killed lactobacilli, whereas the cells responded appropriately towards stimulation with *E. coli*, leading to increased NF-κB activation and release of inflammatory mediators. Co-stimulation with viable lactobacilli and heat-killed *E. coli *did however result in increased NF-κB activation compared to cells challenged with *E. coli *alone (Figure 3A). This NF-κB induction was beyond an eventual additive effect, representing a synergistic action on NF-κB activation. On the protein level, co-stimulation influenced the release of all studied inflammatory mediators. The TNF release was increased by a factor of two to three, while IL-6 and CXCL8 levels were reduced compared to those found during *E. coli *challenge alone (Figure 3B).

NF-κB activation was significantly reduced when bladder cells were exposed to heat-stable cell wall components of lactobacilli (Figure 3A), indicating that potentiation was mediated by compound(s) released during the growth of *L. rhamnosus *GR-1.

### *L. rhamnosus *GR-1 and GG augmented NF-κB to different levels

**Figure 4:**
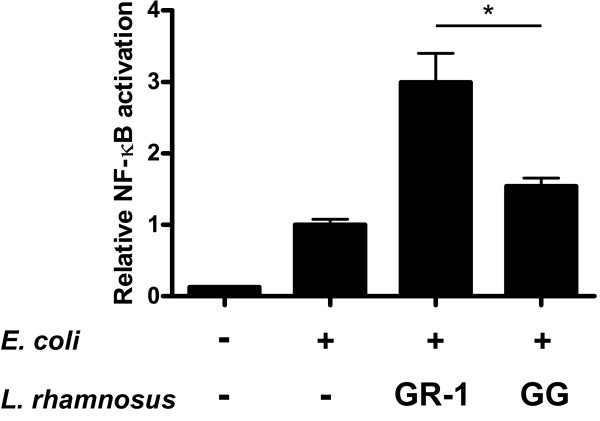
**NF-κB augmentation by two different *L. rhamnosus *strains**. Bladder cells were co-stimulated with heat-killed *E. coli *and viable *L. rhamnosus *GR-1 or *L. rhamnosus *GG for 24 h (*n *= 4). An asterisk denotes significant difference between the two groups (*p*-values < 0.05).

*Lactobacillus rhamnosus *GG, a well-studied immunomodulatory strain used for gastrointestinal disorders, was chosen to compare NF-κB augmenting abilities. Both *L. rhamnosus *GR-1 and GG had the ability to potentiate *E. coli *induced NF-κB activation (Figure 4). While *L. rhamnosus *GG induced NF-κB twofold, *L. rhamnosus *GR-1 showed a three- to fourfold induction of NF-κB compared to cells that had no lactobacilli added.

### *L. rhamnosus *GR-1 modified TLR4 expression on bladder cells

**Figure 5:**
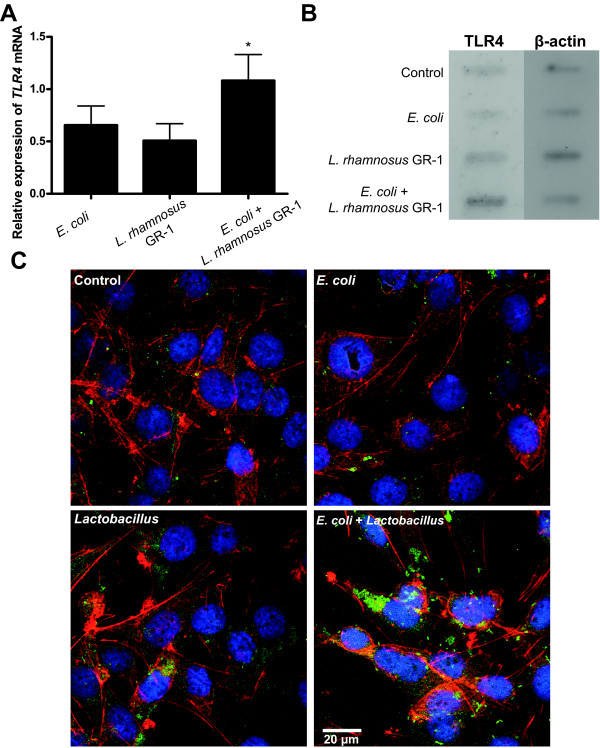
**TLR4 expression in bladder cells after *Lactobacillus *stimulation**. (**A**) *TLR4 *qPCR from cells co-stimulated for 3 h with *E. coli *and *L. rhamnosus *GR-1 (*n *= 3). (**B**) A native immunoblot of TLR4 protein after 24 h stimulation. (**C**) Confocal microscopy of TLR4 protein (green pixels) after stimulation of T24 cells. The cells were also stained with DAPI (DNA stain) and Alexa555 phalloidin (actin stain) and pseudo-colored blue and red, respectively. Immunoblot and confocal images are representative data from two or more separate experiments. Bars labeled with an asterisk are significantly different from control cells (*p*-values < 0.05).

TLR4 is a crucial protein in the detection of *E. coli *by epithelial cells, therefore we proceeded by analyzing the levels of TLR4 in bladder cells treated with heat-killed *E. coli *and *L. rhamnosus *GR-1. Co-stimulated bladder cells showed increased expression of *TLR4 *mRNA compared to cells stimulated with *E. coli *or lactobacilli alone (Figure 5A). Furthermore, immunoblotting using native proteins showed high band intensity in co-stimulated cells suggesting higher TLR4 protein content compared to all other groups (Figure 5B). The effect on TLR4 protein levels was further characterized using confocal laser microscopy. Control cells and cells stimulated with only *E. coli *or lactobacilli showed no or low TLR expression, whereas cells co-stimulated with both *E. coli *and *L. rhamnosus *GR-1 demonstrated a substantial increase in the amount of TLR4 protein (Figure 5C).

### Polymyxin B suppressed NF-κB augmentation

**Figure 6:**
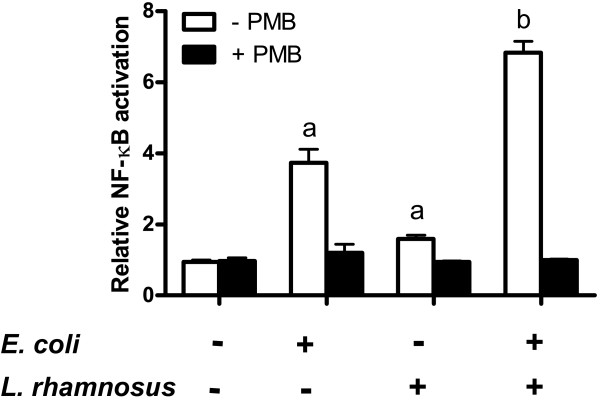
**NF-κB potentiation is TLR4 dependant**. Polymyxin B (PMB) was added to cell culture before stimulation to inhibit TLR4 activation. Cells were stimulated with heat-killed *E. coli *and viable *L. rhamnosus *GR-1 for 24 h (*n *= 3). Bars labeled "a" are significantly different from control and "b" significantly different from *E. coli *stimulated cells (*p*-values < 0.05).

We continued to characterize the role of TLR4 in NF-κB activation by co-stimulation with heat-killed *E. coli *and lactobacilli. The TLR4 activation in bladder cells was inhibited by pretreatment with polymyxin B, a known inhibitor of LPS-induced TLR4 activation, and thereafter stimulated by *E. coli *and *L. rhamnosus *GR-1 (Figure 6). Polymyxin B significantly inhibited NF-κB activation in cells challenged with both *E. coli *and lactobacilli although it had no significant effect on NF-κB activation in resting cells and on lactobacilli treated cells. The increased NF-κB activation observed during co-stimulation was completely lost after polymyxin B treatment, demonstrating the involvement of LPS and TLR4.

## Discussion

Activation of NF-κB during infection has a profound effect on the expression of multiple targets which guide the maturation of immune responses against invading pathogens [22]. Recently, much attention has been given to the immunomodulatory activities of the microbiota and various probiotic organisms. Studies have shown a *L. plantarum *probiotic to be effective at modulating immunity through NF-κB and MAP kinase signaling in a number of cell types including mucosal epithelial cells [23]. In this study we showed the immunomodulatory effects of a urogenital probiotic, *L. rhamnosus *GR-1 on human bladder cells. In order to activate the urothelial cell defense mechanisms in a way that resembles the response during a UTI, including NF-κB and cytokine release, we challenged the cells with heat-killed *E. coli*. Although only live bacteria are active in the infection process, we wanted to reduce the microbe-to-microbe signaling present between viable bacteria as well as the effects of *E. coli *metabolites on cell cultures [24]. Our results showed that bladder cells challenged with heat-killed *E. coli *and subjected to stimulation with *L. rhamnosus *GR-1 exhibited increased NF-κB activation and TNF release.

The finding that *L. rhamnosus *does indeed have immunomodulatory properties is not new per se, but most previous experiments have been done using immune cells [20,25]. Adjuvant properties of *Lactobacillus *species have been demonstrated in several *in vivo *models. An *L. casei *strain boosted immunoglobulin (Ig)A secretion in a mouse model of *Salmonella typhimurium *infection [26]. Another effectively potentiated IgG responses after subcutaneous vaccination of chickens towards Newcastle disease virus and infectious bronchitis virus [27]. Collectively, these studies provide evidence that lactobacilli can be used for potentiating immune responses *in vivo*. Nevertheless, although TNF was upregulated by *L. rhamnosus *GR-1 treatment, anti-inflammatory properties of lactobacilli are well established [25]. In our study, both IL-6 and CXCL8 were modulated differently from TNF, where both were down-regulated after lactobacilli treatment of *E. coli*-challenged cells. These effects might represent an alternative influence of *L. rhamnosus *GR-1 on epithelial immune function, guided by transcription factors other than NF-κB, such as MAP kinase/AP-1 pathways or post-transcriptional regulation of NF-κB-regulated genes. Another possibility is that *L. rhamnosus *GR-1 produces substances that can interfere with cytokine release from the cell or cytokine stability in the extracellular space.

Probiotic health benefits have been shown to be somewhat strain specific. In this study, we showed that two strains exhibit different abilities to increase activation of NF-κB. *L. rhamnosus *GG elicited a weaker potentiation of *E. coli*-induced NF-κB activation than *L. rhamnosus *GR-1. Heat-killed preparations of *L. rhamnosus *GR-1 marginally augmented NF-κB, in a manner similar to using viable *L. rhamnosus *GG (below twofold). It is possible this augmentation is due to surface-associated structures shared by both strains. Lactobacilli surface components have previously been shown to modulate NF-κB in a contact-dependent manner [17]. T24 cells express TLR2, and can recognize lipoteichoic acid (LTA) found on the surface of lactobacilli with increased NF-κB activation as a consequence [28]. However, since heat-killed lactobacilli only slightly induced NF-κB activation that is not a likely mechanism given that LTA is anchored to the Gram-positive cell wall. A more probable mechanism is that products released during bacterial growth are responsible for the NF-κB augmentation by *L. rhamnosus *GR-1. We have previously shown that spent culture supernatant from *L. rhamnosus *GR-1 can augment NF-κB activation in *E. coli*-challenged T24 cells [29]. There are no published studies on the identity of the secreted proteins from *L. rhamnosus *GR-1. However *L. rhamnosus *GG is known to release a small number of proteins during growth, none of which have an established immunomodulatory effect [30]. A comparison of secretory proteins from the two strains might help explain the differences in terms of immune potentiation.

The role of TLR4 was evaluated by blocking LPS binding to the receptor using polymyxin B, which eliminated the observed NF-kB potentiation. We initially saw that expression of TLR4 at genetic and protein levels was increased during co-stimulation compared to controls, or during individual stimulation with *E. coli *or lactobacilli. Although TLR4 has LPS as a natural ligand, other *E. coli *components such as pili have been shown to be able to activate TLR4. However, in this study, polymyxin B completely inhibited NF-κB activation in *E. coli *stimulated cells, therefore pili or other surface structures could not have contributed to this effect [31]. We consider that an increased number of TLR4 present on the cell facilitated activation by ligands on *E. coli *and lactobacilli alike.

TLRs are important in UTI disease progression, as shown in C3H/HeJ mice with a mutation in the *Tlr4 *gene. After an *E. coli *infection, these mutant mice have problems removing the pathogens from their urinary tract [32]. A recent study scoring TLR4 expression levels in healthy control subjects and UTI patients showed that the latter have a lower TLR4 expression than healthy controls [9]. This important feature of TLR4 is consistent with the effect that certain *E. coli *strains expressing immunomodulatory compounds have on TLR signaling and NF-κB activation. The effect of lactobacilli on NF-κB, TNF and TLR4 represents one possibility that increases the urothelial immune cell responses. This augmentation might facilitate early detection and clearance of pathogens.

As defined by FAO/WHO, probiotic microbes must be alive when administered in order to confer health benefits [33]. The *in vitro *effects on NF-κB augmentation has been reported to be dependent on lactobacilli viability, since after heat-killing they only had a marginal effect on NF-κB activation in co-stimulation experiments with *E. coli*. This supports modulation of NF-κB as a potential probiotic mechanism. The ability of probiotic lactobacilli to interfere with UPEC colonization in the vagina, and thereby the pathogens' ascension into the bladder, could therefore involve immunomodulatory activity, specifically via NF-κB activation.

## Conclusions

The main cause of UTI is ascending *E. coli *that colonizes the vagina, urethra then bladder. To remove unwanted pathogens, the urothelial cells of the mucosa carry specific receptors, such as TLR4 that can recognize the most common Gram-negative species. Once these receptors bind the cognate bacterial ligand, the epithelial cells respond by producing a range of compounds including cytokines that are strongly regulated by the NF-κB transcription factor. The present *in vitro *study showed that this immune activation could be amplified by probiotic *L. rhamnosus *GR-1. Moreover, augmentation of NF-κB was accompanied by an increase in inflammatory TNF expression. The important recognition molecule TLR4 was found to be up-regulated by *L. rhamnosus *GR-1 on both mRNA and protein level in cells concomitantly challenged with *E. coli*. Moreover, the blocking agonist binding to TLR4 completely inhibited the augmentation of NF-κB by *L. rhamnosus *GR-1. Due to the importance of TLR4 in the process of pathogen clearance we suggest that this represents a pathway in which probiotic immunomodulatory lactobacilli work to increase immunity and prevent infections.

## Methods

### Cell culture

The T24 human bladder carcinoma cell line (ATCC HTB-4) was cultured in RPMI 1640 (Hyclone) supplemented with 2.05 mM of L-glutamine and 10% fetal bovine serum (FBS; Hyclone) at 37°C with 5% CO_2 _in a humidified environment.

### Bacterial strains and growth conditions

*L. rhamnosus *GR-1 (urethral isolate) and GG (intestinal isolate) were cultured on de Man Rogosa Sharp (MRS) agar (Difco) anaerobically using anaerobic packs (BD) at 37°C for 24 h under static condition. For cell culture challenge, lactobacilli were grown from a 1% inoculum in MRS broth for 24 h followed by washing and resuspending in the original volume with phosphate buffered saline (PBS; pH 7.4). Uropathogenic *E. coli *GR12 was grown in Luria-Bertani (LB) medium (Difco) at 37°C and constant shaking. Heat-killed bacteria were prepared by washing cultures in PBS and heating at 70°C for 1 h followed by plating 100 μl on the respective growth medium (MRS or LB) to confirm loss of viability. Heat-killed *L. rhamnosus *GR-1 and *E. coli *were stored at -20°C until used for cell challenge.

### Transfection and luciferase reporter assay

To detect activation of NF-κB, a luciferase vector composed of multiple κB enhancer regions followed by the firefly luciferase gene was used (pNFκB-Luc, Clontech). A vector with constitutively active *Renilla *luciferase (pRL-CMV, Promega) was chosen as internal control. One day prior to transfection, approximately 0.5 × 10^5 ^cells per well were seeded in a 24-well format. Transfection was performed for 6 h using 1.5 μl/well of Lipofectamine 2000 (Invitrogen), 0.54 μg/well of pNFκB-Luc and 0.06 μg/well of pRL-CMV. Lipofectamine 2000 and plasmids were diluted in serum-free Opti-MEM (Invitrogen) during preparation of DNA-liposome complexes. All plasmids were isolated by an endofree plasmid isolation kit (Macherey-Nagel) according to the manufacturer's instructions. Luciferase was detected using the dual-luciferase reporter assay system (Promega) and a Turner TD20/20 luminometer (Turner biosystems) set to 10s measurement with an initial 2s delay. Transcription factor activation was expressed as relative NF-κB activation, defined as the ratio between firefly luciferase and *Renilla *luciferase activity. Ratios were normalized against either non-stimulated control cells or cells stimulated with *E. coli*. The difference between means was tested statistically by using Student's t-test, with the limit for statistical significance set to p-values < 0.05.

### Epithelial cell line challenge

T24 bladder cells transfected with luciferase vectors (pNFκB-Luc and pRL-CMV) were challenged for 24 h in a 24-well plate format with 2 × 10^7 ^cfu/ml of viable or the equivalent number of heat-killed lactobacilli (*L. rhamnosus *GR-1 or GG). For activation of NF-κB, as well as cytokine and chemokine release, epithelial cells were stimulated with heat-killed *E. coli *(10^8 ^cfu/ml). Cell culture supernatants for ELISA were collected from challenge experiments using non-transfected cells and stored at -20°C until use. For qPCR, cells were stimulated in the same way although all experiments were done in 6-well plates (with proportional increase in number of cells and bacteria) for increased amounts of RNA. Cell viability was determined by staining dead cells using propidium iodide followed by flow cytometry (Cytomics FC500, Beckman Coulter). To inhibit agonist activation of TLR4 in T24 cells, transfected cells were exposed to Polymyxin B (Invivogen), which effectively binds to LPS and thereby inhibits TLR4 activation, at a concentration of 50 μg/ml for 1 h prior to the experiment and subsequently challenged with bacteria, as previously described.

### Enzyme-linked immunosorbent assays

TNF, IL-6 and CXCL8 levels were determined by BD ELISA sets (BD Biosciences) according to the manufacturer's instructions. A volume of 100 μl of capture antibody (diluted 1:250 coating buffer) was added to each well of a 96-well ELISA microplate (Nunc) and allowed to bind overnight at 4°C. Wells were washed three times with PBST (PBS pH 7.0 with 0.05% Tween-20) and blocked with PBS supplemented with 10% heat-inactivated FBS (HyClone) for 1 h in room temperature after which the wells were washed three times with PBST. Tissue culture medium from challenged cells was briefly centrifuged to remove cell debris and 100 μl of the supernatant or standard was added to the wells and incubated at room temperature for 2 h. After washing five times with PBST, 100 μl detection antibody:HRP conjugate (diluted 1:250 in PBS with 10% heat-inactivated FBS) was added to the wells and incubated for 1 h at room temperature. After extensive washing (seven times using PBST), 100 μl of H_2_O_2_/3,3',5,5'-tetramethylbenzidine prepared according to the manufacturer's instructions (TMB substrate reagent set, BD Biosciences) was added to each well and incubated at room temperature for 30 min in the dark. The reaction was stopped with 2 N H_2_SO_4 _and absorbance read at 450 nm using a Multiskan MS plate reader (Labsystems). Difference between means was tested statistically by using the Student's t-test, with the limit for statistical significance set to *p*-values < 0.05.

### Quantitative polymerase chain reaction

Total RNA was extracted using the Nucleospin RNA II Kit (Macherey-Nagel) with a DNase treatment step. cDNA was synthesized from 1 μg of extracted total RNA using qScript cDNA Synthesis Kit (Quanta Biosciences). Quantitative real time PCR was performed using Perfecta SYBR Green Fastmix on a Stratagene MX3000 QPCR system (Agilent Technologies) according to the manufacturer's instructions. Primers were designed to bind to different exons within the genes thereby avoiding risk of genomic DNA amplification. The primers had a Tm = 60°C with the following sequences: GAPDH: 5' CCGTCTAGAAAAACCTGCCA 3' and 5' TGTGAGGAGGGGAGATTCAG 3'; TLR4: 5' CTGAGCTTTAATCCCCTGAGGC 3' and 5' AGGTGGCTTAGGCTCTGATATGC 3'. All reactions were run in triplicate. Results were analyzed using MxPro QPCR software (Agilent Technologies) and statistics were performed on adjusted ratios using a non-parametric Mann-Whitney U test. The limit for statistical significance was set to *p*-values < 0.05.

### Immunoblot

Cells were grown and challenged as previously described in a six-well format, and thereafter lysed using RIPA buffer. Immunoblotting of cell lysate onto a PVDF membrane (Amersham Biosciences) was performed using vacuum. Unbound PVDF sites were blocked with blocking buffer (Tris-buffered saline, TBS, containing 0.05% Tween-20 and 1% BSA) for 1 h. Blotted membrane was incubated in primary antibody solution (anti-TLR4, clone HTA125; BD Biosciences or anti-β-actin, clone AC-15; Sigma-Aldrich) resuspended in blocking buffer at a concentration of 1 μg/ml (anti-TLR4) or 10,000 times dilution (anti-β-actin) for 1 h at room temperature and thereafter washed 3 times for 5 min in wash buffer (TBS and 0.05% Tween-20). For visualization, the membrane was incubated with the secondary antibody (anti-mouse IgG HRP-conjugated, GE Healthcare) at a 10,000 times dilution for 1 h in room temperature. The membrane was washed 4 times for 5 min using wash buffer before the addition of chemiluminescent substrate (Supersignal west pico, Pierce). Luminescence was detected using a photographic film (GE Healthcare). In order to use the loading control antibody (anti-β-actin), the membrane was stripped using a mild stripping agent (200 mM glycine, 0.01% (v/v) Tween-20, 3.5 mM SDS, pH 2.2).

### Confocal microscopy

Cells were grown in a 6-well format on cover slips overnight and challenged as described above. The cells were washed twice in PBS and fixed in 4% paraformaldehyde for 10 min followed by washing twice for 5 min in PBS. Cells were permeabilized with PBS containing 0.25% Triton X-100 (PBST) for 10 min and washed 3 times with PBS prior to blocking with 1% bovine serum albumin in PBST (PBST-BSA) for 30 min. Primary antibody (anti-TLR4, clone HTA125, BD Biosciences) was added to cells at a concentration of 0.5 μg/ml in PBST-BSA and incubated overnight at 4°C. Cells were washed 3 times in PBS and thereafter incubated for 1 h at room temperature with anti-mouse FITC antibody (BD Biosciences) diluted in PBST-BSA at a concentration of 0.5 μg/ml. FITC-staining was followed by washing with PBS and subsequent staining of actin using Alexa555 phalloidin (Molecular probes) for 30 min at room temperature. The cells were rinsed with PBS twice and incubated with a 30 nM DAPI solution for 1 min before mounting onto glass slides. Fluorescence was observed through a Fluoview 1000 scanning confocal laser microscope with the FV10-ASW software (Olympus).

## Competing interests

The authors declare that there are no competing interests.

## Authors' contributions

MK participated in the study design, carried out majority of the experimental work and writing of the manuscript. NS was responsible for the qPCR analysis. GR participated in the study conception and revising of the manuscript. JJ conceived and participated in the study design, coordinated the study and writing of the manuscript. All authors read and approved the final manuscript.
